# Dietary patterns and cardiovascular diseases among Chinese high-risk population aged 35 years and older: A 6-year cohort study

**DOI:** 10.1016/j.crfs.2024.100960

**Published:** 2024-12-16

**Authors:** Shanshan Chen, Shiyun Hu, Sijie Shen, Jialin Zhang, Xiaohui Xu, Ming Yu, Yu Xia, Qiang Cai, Wei Yu, Anni Lu, Ziqi Mia Li, Rasika Gunarathne, Jun Lu

**Affiliations:** a Yangtze Delta Region Institute of Tsinghua University, Zhejiang, 314006, China; b Zhejiang Provincial Center for Cardiovascular Disease Control and Prevention, Zhejiang Hospital, Hangzhou, 310013, China; c Pinehurst School, Albany, Auckland, 0632, New Zealand; d Saint Kentigern College, Pakuranga, Auckland, 2010, New Zealand; e Auckland Bioengineering Institute, University of Auckland, Auckland, 1142, New Zealand

**Keywords:** Dietary pattern, Cardiovascular disease, Factor analysis, Disease risk

## Abstract

This study aims to investigate the dietary patterns of Chinese individuals aged 35 years and older who are at high risk of cardiovascular disease (CVD) and to explore the correlation between these dietary patterns and the risk of CVD. A total of 28,747 high-risk participants in China PEACE in Zhejiang Province from 2014 to 2019 were included in the analysis Dietary data were obtained using the Food Frequency Questionnaire, and dietary patterns were extracted through factor analysis. Cox regression was used to examine the relationship between the dietary patterns and CVD risk in the high-risk groups. Four dietary patterns were identified. The “Bean, egg, milk and pickle” dietary pattern was associated with an increased risk of CVD (HR = 1.29; 95% confidence interval (CI):1.09, 1.54; p＜0.05), after adjusting for confounders. In contrast, the “Seafood and animal meat”, “Wheat and coarse cereals”, and “Rice and vegetable” dietary patterns did not show a significant impact on CVD risk. These findings provide valuable insights for dietary guidance in high-risk groups and have significant implications for the prevention and management of CVD.

## Highlights

• We conducted a prospective cohort study with 28474 participants.
• The study lasted for 6 years.
• “Bean, egg, milk and pickle” dietary pattern is associated with a reduced risk of CVD.
• Other dietary patterns have no significant association with CVD.

## Graphical abstract

**Figure:**
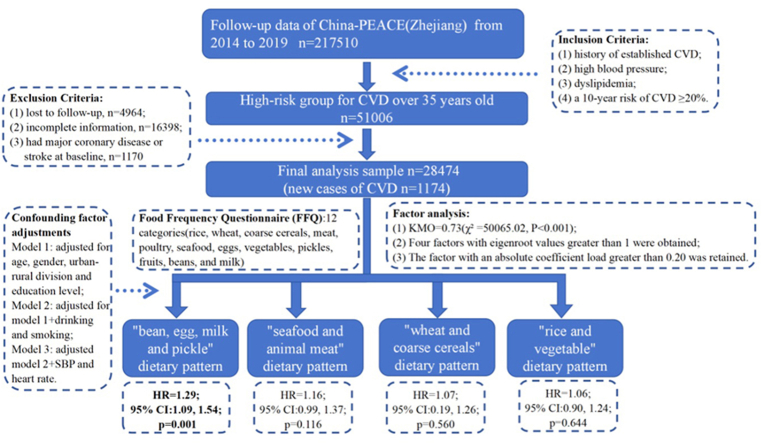

## 1 Introduction

Cardiovascular disease (CVD) has emerged as the leading cause of death and disease burden in China and globally (Vos et al., 2020). According to global disease burden research data, there were 523 million CVD patients worldwide by 2019, with 18.57 million deaths attributed to CVD, and the disability-adjusted life years caused by CVD reached 393 million years. Notably, China experiences the highest CVD-related mortalit, accounting for 4.58 million deaths, or 43.04% of all mortalities (Roth et al., 2020). Projections suggest a more than 50% increase in CVD events in China over the next two decades (Moran et al., 2010), with estimates suggesting up to 20 million myocardial infarctions (MIs) and 30 million strokes annually by 2030 (Wang et al., 2011). This growing health crisis is further amplified by China's aging and increasingly urbanized population, highlighting the importance of addressing cardiovascular health. Evidence suggests that a healthy dietary pattern plays a crucial role in maintaining a healthy weight and reducing the risk of CVD and its associated risk factors. Dietary patterns rich in red meat, fast food, sweets, oil, and salt have been linked to an increased risk of CVD, largely due to their high energy, carbohydrate, fat, and sodium content, coupled with low potassium and calcium levels (Lara et al., 2019; Farmaki et al., 2019; Whitton et al., 2018). Conversely, diets that emphasize vegetables, fruits, nuts, whole grains, and legumes have beenshown to provide protective effects against CVD (Lara et al., 2019; Farmaki et al., 2019; Whitton et al., 2018)^.^ These dietary patterns are typically low in calories, fat, carbohydrate and sodium, while being rich in dietary fiber, vitamins and minerals (calcium, magnesium, potassium, selenium, etc.) (Xu et al., 2017). Furthermore, protein-rich diets have been found to lower blood pressure (Teunissen-Beekman et al., 2012), potentially through the action of antihypertensive peptides derived from food protein, or by inhibiting the effect of angiotensin on Ca^2+^ transport via amino acids such as taurine. Various dietary patterns, including the Healthy US-Style Eating, Healthy Mediterranean-Style eating, Healthy Vegetarian Eating pattern (Freeman et al., 2017; −2020 Dietary Guidelines for Americans, 2015) and the DASH diet (Filippou et al., 2020; Fung et al., 2008) have been associated with an 19–28% reduction in CVD mortality (George et al., 2014; Harmon et al., 2015; Liese et al., 2015; Reedy et al., 2014). The Spanish PREDIMED (PREvención con DIeta MEDiterránea) trial, which emphasized components like olive oil and nuts, demonstrated a reduction in major CVD events including stroke, myocardial infarction, and cardiovascular mortality (Estruch et al., 2013). Daily consumption of fresh fruits has also been associated with decrease risks of cardiometabolic diseases in Chinese populations (Du et al., 2016, 2017). This protective effect is likely due to the high content of dietary fiber, potassium, vitamins, and antioxidants found in fruits, which contribute to improved lipid profiles, enhanced insulin sensitivity, reduced blood pressure, better regulation of hemostasis, and neutralizion of oxidative stress (Alissa and Ferns, 2017).

Compared to traditional studies focusing on single foods or nutrients, examining overall dietary patterns offers a more practical approach for developing nutritional interventions, such as dietary guidelines, which are easier for the public to understand and apply, thus carrying greater public health significance. However, research on the relationship between dietary patterns and cardiovascular disease (CVD) in China remains limited, with most studies focusing on adult populations and lacking data on high-risk groups for CVD. Currently, dietary patterns are commonly classified using three approaches: a prior, a posterior and mixed method. Among these, the a posteriori method stands out for its ability to capture dietary patterns that reflect local characteristics, making it valuable for exploring regional food culture, dietary diversity, accessibility, and disease prevalence across various populations. This study aimed to identify dietary patterns within high-risk groups in China and assess their association with cardiovascular diseases. By elucidating these relationships, the study seeks to offer actionable guidance for the prevention and management of CVD, contributing to public health efforts to ruduce the national burden of CVD.

## 2 Methods

### 2.1 Subjects and methods of data collection

This study utilized 6-year follow-up data from the China-PEACE study in Zhejiang Province from 2014 to 2019 (Lu et al., 2016a). Participants, aged 35–79, were selected based on the cardiovascular risk prediction charts developed by the WHO/International Society of Hypertension (ISH) (World Health Organization, 2007a). The China-PEACE project represents the first large-scale population-based screening initiative in China designed to identify individuals at high risk for CVD. "High CVD risk" was defined by meeting one or more of the following criteria: (1) history of established CVD, (2) high blood pressure, (3) dyslipidemia, and (4) a 10-year risk of CVD ≥20% (World Health Organization, 2007b). Among the baseline participants, 51006 high-risk groups (23.45%) completed the required questionnaires, provided lifestyle (eg, smoking, drinking, diet) and medical history information, completed a food frequency questionnaire (FFQ), underwent physical examination, and provided fasting blood samples (Lu et al., 2016b). Of these, 4964 individuals lost to follow-up, 16398 had incomplete information, and 1170 participants with major coronary disease or stroke at baseline were excluded (based on self-report and previous diagnosis). Consequently, 28474 participants were included in the final analysis. These participants were followed for morbidity and mortality outcomes until 2022. Ethical approval for this study was granted by the Zhejiang Hospital Medical Ethics Committee (No 27 K, 2019). All participants provided written informed consent, documented either by thumbprint or signature.

### 2.2 Dietary intake assessment

A balanced diet is the cornerstone of health, and dietary nutritional factors play a critical role in the rising prevalence of chronic diseases. The relationship between dietary patterns and chronic diseases has become a significant focus in recent research, both in understanding etiology and in developing strategies for prevention and treatment. In this study, dietary data were derived from follow-up questionnaires administered by the China-PEACE project (Lu et al., 2016a). A standardized food frequency questionnaire was used to collect the frequency of various food items consumed over the past month by high-risk participants (Lu et al., 2016a). The Food Diet questionnaire categorized foods into 12 groups aligned with the survey's objectives: rice, wheat, coarse cereals (e.g. millet, corn, and sorghum), meat, poultry, seafood, eggs, vegetables, pickles, fruits, beans, and dairy. Respondents participated in face-to-face interviews, reporting their food inake frequency over the past month and filling out questionnaires(Lu et al., 2016a). The frequency of consumption for each food group involved five categories: never or rarely, 1–3 days/month, 1–3 days/week, 4–6 days/week and daily. These frequencies levels were then numerically coded as 5, 4, 3, 2, and 1, respectively.

### 2.3 Factor analysis

Dietary patterns analysis has emerged as a complementary method to studying the relationship between diet and human health (Kant, 2004). Among the various methods available, factor analysis is the most commonly employed. This technique identifies dietary patterns by examining the correlation between food groups. By reducing a wide range of food variables into a smaller set of variables that reflect the main dietary characteristics of the study subjects. Factor scores for each individual can then be used to analyze the relationship between dietary patterns and health outcomes (Zhu et al., 2016). Dietary patterns in this study were derived from the consumption data of 12 food groups using factor analysis with the principal component method (Diabetes Branch of Chinese Medical Associatio n et al., 2019). Orthogonal rotation (maximum variance method) was applied to enhance the interpretability and independence of factors. The number of factors (dietary patterns) was determined based on criteria including an eigenvalue greater than 1, scree plot analysis, interpretability of factors, and the variance explained by each factor, which was required to exceed 50%. Dietary patterns were named according to the food groups with high absolute loadings on each factor. Factor scores for each dietary pattern and participant were calculated by summing the consumption of each food group, weighted by its respective factor loading. In order to understand the adherence of each dietary pattern, scores were divided intoquartiles, with Q_1_ representing the lowest adherence and Q_4_ representing the highest adherence.

### 2.4 Covariates evaluations

A baseline survey was employed to collect comprehensive information on the sociodemographic characteristics, lifestyle behaviors, and medical history of the participants. The survey questions were adapted from previous population-based epidemiological studies conducted in China (Chen et al., 2011; Strauss et al., 2010). Key sociodemographic variables included age at recruitment, sex, education level, marital status, and residence in urban or rural areas. Lifestyle behaviors assessed included smoking and alcohol consumption. Medical history, including diagnosed CVD, was recorded while anthropometric measurements (body weight and height) and blood glucose levels (random and fasting) were assessed by trained staff following standardized protocols (Lu et al., 2016a).

Definition of related variables: (1) CVD: Self-reported CVD must be clearly diagnosed by a doctor; (2) Drinking: Refers to any alcohol consumption within the past 30 days; (3) Smoking: Includes both current and past smoking behaviors; (4) Body Mass Index (BMI): Categorized according to the Chinese guidelines for the prevention and control of overweight and obesity in adults (Department of Disease Control and Ministry of Health of the People's Republic of China, 2006), BMI.

### 2.5 Endpoint ascertainment

The China-PEACE study linked participants to local disease and death registries, cross-checked against a password-protected database at the China National Center for Cardiovascular Disease (NCCD), and periodically carried out active follow-up to monitor participants’ vital signs and disease status (Lu et al., 2016a). Clinicians, unaware of the baseline characteristics, coded diseases based on the International Classification of Diseases, 11th Revision (ICD-11). The primary endpoints included major coronary events (L1-BA8), stroke events (8B11), and all-cause mortality. The person-year of follow-up refers to the time duration between the date when the respondent entered the queue and the time when he/she was diagnosed with CVD, death, or the end of follow-up.

### Statistical analysis

Normally distributed quantitative data were described as mean ± standard deviation (x ± s), and *t*-test or analysis of variance were used for comparison between groups. For quantitative data with a skewed distribution, median (M) and interquartile range (P25, P75) were used for description, and the Mann-Whitney *U* test was employed for group comparisons. Categorical data were presented as frequencies and percentages, with chi-square tests used for group comparisons. The baseline characteristics of the participants were stratified by quartiles of adherence to the four identified dietary patterns. Trend associations were evaluated across quartiles using linear regression for continuous and categorical variables, respectively. Cox proportional hazards models estimated the hazard ratio (HR) and 95% confidence interval (95% CI) for each outcome based on the quartile of adherence to each dietary pattern, using the lowest quartile as the reference. Adjustments for potential confounders were made based on baseline characteristics across dietary patterns: Model 1 adjusted for age, gender, urban-rural division, and education level; Model 2 additonally adjusted for smoking and drinking status; Model 3 further adjusted for systolic blood pressure (SBP) and heart rate. Age, SBP, and heart rate were treated as continuous variables, while other covariates were treated categorically. For sensitive analysis, participants with follow-up periods shorter than two years were excluded to minimize potential reverse causality. All statistical analyses were performed using SPSS 19.0.

## 3 Results

### 3.1 Baseline characteristics of study subjects

Table 1 shows the baseline characteristic data of the study population. The data indicate that a total of 28,474 participants were included in the study, with an average age of 59 years. Among them, 13318 were male (46.77%), and the average BMI was 24.91 kg/m^2^. Over the follow-up period, 1174 participants developed CVD, resulting in a crude incidence rate of 7.40/1000 person-years. Baseline characteristics showed significant differences (p < 0.05) between the CVD and non-CVD groups for most variables, except for drinking, BMI, diastolic blood pressure (DBP), total cholesterol (TC), high-density lipoprotein (HDL), and triglycerides (TG), where no significant differences (p > 0.05) were observed. Within high-risk groups, individuals who developed CVD were primarily concentrated among lower-educated men residing in rural areas who also had higher rates of smoking and alcohol consumption.

**Table 1:** Baseline characteristics of the study participants [n(%)/M(P25,P75)].

Characteristic	Total n = 28474	Non-CVD n = 27300(95.88)	CVD n = 1174(4.12)	*p*-value
Age (years)	59(52, 66)	59(51, 65)	66(60, 65)	**<0.001**
Male (%)	13318(46.77)	12655(46.36)	663(56.47)	**<0.001**
Urban residents (%)	6221(21.85)	5901(21.62)	320(27.26)	**<0.001**
Education level (%)				
Unknown	220(0.77)	211(0.77)	9(0.77)	**<0.001**
Primary school and below	20213(70.99)	19268(70.58)	945(80.49)	**<0.001**
Junior/senior high school	7722(27.12)	7509(27.51)	213(18.14)	**<0.001**
College and above	319(1.12)	312(1.14)	7(0.60)	**<0.001**
Drinking (%)	6380(22.41)	6104(22.36)	276(23.51)	0.931
Smoking (%)	5932(20.83)	5615(20.57)	317(27.00)	**<0.001**
BMI (kg·m^−2^)	24.9(22.9, 27.1)	24.9(22.9, 27.1)	24.9(22.8, 27.1)	0.697
SBP (mmHg)	162(143, 171)	162(143, 170)	165(153, 170)	**<0.001**
DBP (mmHg)	89(80, 97)	89(80, 97)	89(80, 97)	0.378
Heart rate (beats/min)	76(70, 84)	76(69, 84)	77(70, 84)	**0.004**
TC (mmol/L)	5.09(4.28, 6.21)	5.09(4.28, 6.22)	5.09(4.28, 6.22)	0.129
LDL (mmol/L)	2.87(2.13, 3.61)	2.88(2.13, 3.62)	2.81(2.10, 3.62)	**0.020**
HDL (mmol/L)	1.42(1.11, 1.77)	1.42(1.11, 1.77)	1.45(1.13, 1.77)	0.209
TG (mmol/L)	1.71(1.14, 2.59)	1.70(1.14, 2.59)	1.73(1.18, 2.59)	0.608

Abrreviations: CVD, cardiovascular disease; BMI, body mass index; SBP, systolic blood pressure; DBP, diastolic blood pressure; TC, total cholesterol; LDL, high-density lipoprotein; HDL, high-density lipoprotein; TG, triglycerides.

### 3.2 Types and characteristics of dietary patterns

The results of the exploratory factor analysis indicated that the dietary data were suitable for factor analysis, as evidenced by a Kaiser-Meyer-Olkin (KMO) value of 0.73 and a statistically significant difference in Bartlett's test of sphericity (χ^2^ = 50065.02, p < 0.001). Factor analysis reduced the dimensionality of the dietary data to four factors with eigenroot values greater than 1, specifically 2.77, 1.60, 1.28, and 1.71, cumulatively explaining 56.87% of the total variance. A factor loading threshold of 0.200 was applied, so food groups with absolute loadings exceeding 0.200 were retained in the factor loading matrix, resulting in the identification of four distinct dietary patterns.

The four identified dietary patterns and their characteristics are as follows: The "Bean, egg, milk, and pickle" dietary pattern, accounting for 18.92% of the variance, was characterized by high consumption of eggs, pickles, fruits, beans and milk, with moderate poultry intake. The "Seafood and animal meat" dietary pattern, explaining 14.06% of the variance, involved high consumption of meat, poultry and seafood, with a moderate intake of egg and pickles. The"Wheat and coarse cereals" dietary pattern, accounting for 13.66% of the variance, featured high consumption of wheat and grain, low intakes of fruit, bean, and milk, and negative associations with poultry and pickle. Lastly, the "Rice and vegetable" pattern, explaining 10.23% of the variance, was marked by high consumption of rice and vegetables, with low meat intake (Table 2).

**Table 2:** Components of dietary patterns and factor loadings.

Characteristic	Factor loadings			
	Bean, egg, milk and pickle	Seafood and animal meat	Wheat and coarse cereals	Rice and vegetable
rice	−0.039	0.028	−0.107	**0.722**
wheat	0.124	0.081	**0.813**	0.030
coarse cereals	0.104	0.096	**0.752**	−0.093
meat	0.065	**0.649**	0.147	**0.231**
poultry	**0.451**	**0.642**	**−0.229**	−0.018
seafood	−0.083	**0.775**	0.151	−0.067
egg	**0.653**	**0.344**	−0.020	−0.192
vegetable	0.047	0.055	0.054	**0.743**
pickle	**0.629**	**0.280**	**−0.231**	0.053
fruit	**0.586**	−0.058	**0.378**	0.065
bean	**0.656**	0.026	**0.235**	0.180
milk	**0.655**	−0.179	**0.223**	−0.106

Figures in bold indicate absolute factor loading are more than 0.200.

### 3.3 Baseline characteristic distribution of dietary patterns

The data presented in Table 3, Table 4, Table 5, Table 6 depict the distribution of baseline characteristics of dietary patterns. The four dietary patterns showed statistically significant differences (p < 0.05) in baseline characteristics, including age, gender, place of residence, alcohol consumption, smoking, SBP, and heart rate. In addition, the "Wheat and coarse cereals" dietary pattern was significantly negatively associated with education level (p = 0.045), and was mainly observed among individuals with a junior high school education or lower. For the "Bean, egg, milk and pickle" dietary pattern, participants in group Q_4_ (highest adherence) had fewer urban residents and fewer individuals with unknown education levels and college-level education compared to group Q_1_ (lowest adherence) (Table 3). In the "Seafood and animal meat" dietary pattern, group Q_4_ had fewer males, drinkers, and smokers but a higher proportion of urban residents compared to group Q_1_ (Table 4). For the "Wheat and coarse cereals" dietary pattern, groups Q_3_ and Q_4_ had relatively lower proportions of urban residents and individuals with a college education and above compared to group Q_1_ (Table 5). In the "Rice and vegetable" dietary pattern, group Q_4_ had a higher proportion of urban residents compared to group Q_1_ (Table 6).

**Table 3:** Baseline characteristics of quartile (Q) of CVD high-risk population according to "Bean, egg, milk and pickle" dietary pattern[n(%)/M(P25,P75)].

Characteristic	"Bean, egg, milk and pickle" dietary pattern				
	Q_1_ (n = 7119)	Q_2_ (n = 7284)	Q_3_ (n = 6983)	Q_4_ (n = 7118)	p _trend_
Age (years)	59(51, 65)	59 (51, 65)	60(52, 66)	60(53, 66)	**0.001**
Male (%)	3571(50.16)	3382(46.43)	3179(45.72)	3186(44.76)	**0.001**
Urban residents (%)	2638(37.06)	2179(29.91)	948(13.63)	456(6.41)	**0.001**
Education level (%)					0.064
Unknown	111(1.56)	45(0.62)	50(0.72)	14(0.20)	
Primary school and below	4667(65.56)	4589(63.00)	5031(72.36)	5926(83.25)	
Junior/senior high school	2241(31.48)	2532(34.76)	1794(25.80)	1155(16.23)	
College and above	100(1.40)	118(1.62)	78(1.12)	23(0.32)	
Drinking (%)	1754(24.64)	1742(23.92)	1500(21.57)	1384(19.44)	**0.027**
Smoking (%)	1522(21.38)	1457(20.00%)	1419(20.41)	1534(21.55)	**0.001**
BMI (kg·m^−2^)	24.7(22.8, 26.9)	24.7(22.7, 27.0)	24.9(22.8, 27.1)	25.3(23.2, 27.5)	0.189
SBP (mmHg)	161(142, 170)	162(143, 171)	163(146, 171)	162(144, 171)	**0.001**
DBP (mmHg)	90(81, 99)	89(80, 97)	89(80, 97)	88(79, 96)	0.130
Heart rate (beats/min)	76(69, 83)	76(70, 84)	76(70, 84)	76(69, 84)	**0.001**
TC (mmol/L)	4.84(4.12, 5.79)	5.05(4.28, 6.18)	5.15(4.31, 6.22)	5.34(4.47, 6.48)	0.634
LDL (mmol/L)	2.68(2.06, 3.24)	2.83(2.08, 3.55)	2.87(2.12, 3.64)	2.94(2.29, 4.08)	0.821
HDL (mmol/L)	1.40(1.10, 1.74)	1.45(1.14, 1.80)	1.44(1.11, 1.80)	1.41(1.10, 1.75)	0.564
TG (mmol/L)	1.59(1.06, 2.41)	1.68(1.15, 2.56)	1.75(1.19, 2.64)	1.83(1.21, 2.77)	0.438

Abrreviations: CVD, cardiovascular disease; BMI, body mass index; SBP, systolic blood pressure; DBP, diastolic blood pressure; TC, total cholesterol; LDL, high-density lipoprotein; HDL, high-density lipoprotein; TG, triglycerides.

Q_1_:lowest adherence; Q_2_:second adherence; Q_3_:third adherence; Q_4_:highest adherence.

**Table 4:** Baseline characteristics of quartile (Q) of CVD high-risk population according to "Seafood and animal meat" dietary pattern[n(%)/M(P25,P75)].

Characteristic	"Seafood and animal meat" dietary pattern				
	Q_1_ (n = 7199)	Q_2_ (n = 7392)	Q_3_ (n = 6766)	Q_4_ n = 7117)	p _trend_
Age (years)	59 (51, 65)	58(51, 65)	60(52, 66)	61(52, 67)	**0.001**
Male (%)	3743(51.99)	3774(51.06)	3105(45.89)	2696(37.88)	**0.001**
Urban residents (%)	732(10.17)	2093(28.31)	1839(27.18)	1557(21.88)	**0.001**
Education level (%)					0.061
Unknown	48(0.67)	44(0.60)	85(1.26)	43(0.60)	
Primary school and below	4924(68.40)	5086(68.80)	4781(70.66)	5422(76.18)	
Junior/senior high school	2152(29.89)	2163(29.26)	1813(26.80)	1594(22.40)	
College and above	75(1.04)	99(1.34)	87(1.29)	58(0.81)	
Drinking (%)	2116(29.39)	1905(25.77)	1359(20.09)	1000(14.05)	**0.021**
Smoking (%)	1655(22.99)	1707(23.09)	1402(20.72)	1168(16.41)	**0.001**
BMI (kg·m^−2^)	25.3(23.3, 27.4)	25.0(23.0, 27.3)	24.8(22.7, 27.0)	24.4(22.5, 26.6)	0.210
SBP (mmHg)	161(142, 169)	162(143, 170)	162(144, 171)	163(145, 172)	**0.001**
DBP (mmHg)	88(80, 97)	89(81, 98)	89(80, 97)	88(80, 97)	0.135
Heart rate (beats/min)	75(69, 83)	76(69, 83)	76(70, 84)	77(70, 85)	**0.001**
TC (mmol/L)	5.22(4.36, 6.33)	5.01(4.22, 6.12)	5.07(4.27, 6.18)	5.07(4.27, 6.17)	0.624
LDL (mmol/L)	2.94(2.24, 3.93)	2.82(2.13, 3.52)	2.82(2.09, 3.49)	2.80(2.08, 3.55)	0.838
HDL (mmol/L)	1.42(1.12, 1.75)	1.40(1.09, 1.75)	1.44(1.12, 1.80)	1.43(1.11, 1.79)	0.580
TG (mmol/L)	1.74(1.16, 2.64)	1.64(1.10, 2.51)	1.72(1.15, 2.61)	1.74(1.16, 2.64)	0.456

Abrreviations: CVD, cardiovascular disease; BMI, body mass index; SBP, systolic blood pressure; DBP, diastolic blood pressure; TC, total cholesterol; LDL, high-density lipoprotein; HDL, high-density lipoprotein; TG, triglycerides.

Q_1_:lowest adherence; Q_2_:second adherence; Q_3_:third adherence; Q_4_:highest adherence.

**Table 5:** Baseline characteristics of quartile (Q) of CVD high-risk population according to "Wheat and coarse cereals" dietary pattern[n(%)/M(P25,P75)].

Characteristic	"Wheat and coarse cereals" dietary pattern				
	Q_1_ (n = 7119)	Q_2_ (n = 7118)	Q_3_ (n = 7119)	Q_4_ (n = 7118)	p _trend_
Age (years)	59(52, 66)	59(51, 66)	59(52, 65)	60(52, 66)	**0.001**
Male (%)	3386(47.56)	3493(49.07)	3277(46.03)	3162(44.42)	**0.001**
Urban residents (%)	2319(32.57)	2328(32.71)	1014(14.24)	560(7.87)	**0.001**
Education level(%)					**0.045**
Unknown	48(0.67)	77(1.08)	35(0.49)	60(0.84)	
Primary school and below	4802(67.45)	4842(68.02)	5214(73.24)	5355(75.23)	
Junior/senior high school	2140(30.06)	2114(29.70)	1811(25.44)	1657(23.28)	
College and above	129(1.81)	85(1.19)	59(0.83)	46(0.65)	
Drinking (%)	1484(20.85)	1632(22.93)	1620(22.76)	1644(23.10)	**0.022**
Smoking (%)	1455(20.44)	1569(22.04)	1486(20.87)	1422(19.98)	**0.001**
BMI (kg·m^−2^)	25.1(23.0, 27.2)	24.9(22.8, 27.1)	24.8(22.8, 27.0)	24.8(22.8, 27.0)	0.215
SBP (mmHg)	162(143, 170)	162(143, 170)	162(144, 171)	162(143, 171)	**0.001**
DBP (mmHg)	88(80, 97)	90(81, 98)	89(80, 97)	88(80, 97)	0.150
Heart rate (beats/min)	76(69, 84)	76(70, 84)	76(70, 84)	76(70, 84)	**0.001**
TC (mmol/L)	5.19(4.34, 6.40)	4.95(4.20, 5.99)	5.13(4.32, 6.26)	5.09(4.29, 6.18)	0.620
LDL (mmol/L)	2.94(2.14, 3.78)	2.78(2.10, 3.34)	2.92(2.14, 3.71)	2.85(2.14, 3.69)	0.822
HDL (mmol/L)	1.44(1.12, 1.80)	1.42(1.11, 1.77)	1.43(1.12, 1.78)	1.41(1.10, 1.73)	0.548
TG (mmol/L)	1.77(1.20, 2.68)	1.66(1.10, 2.50)	1.73(1.16, 2.62)	1.68(1.12, 2.59)	0.463

Abrreviations: CVD, cardiovascular disease; BMI, body mass index; SBP, systolic blood pressure; DBP, diastolic blood pressure; TC, total cholesterol; LDL, high-density lipoprotein; HDL, high-density lipoprotein; TG, triglycerides.

Q_1_:lowest adherence; Q_2_:second adherence; Q_3_:third adherence; Q_4_:highest adherence.

**Table 6:** Baseline characteristics of quartile (Q) of CVD high-risk population according to "Rice and vegetable" dietary pattern[n(%)/M(P25,P75)].

Characteristic	"Rice and vegetable" dietary pattern				
	Q_1_ (n = 7121)	Q_2_ (n = 7119)	Q_3_ (n = 7116)	Q_4_ (n = 7118)	p _trend_
Age (years)	60(52, 66)	60(52, 66)	59(51, 66)	59(51, 66)	**0.001**
Male (%)	3477(48.83)	3236(45.46)	3270(45.95)	3335(46.85)	**0.001**
Urban residents (%)	1575(22.12)	1260(17.70)	1299(18.25)	2087(29.32)	**0.001**
Education level (%)					0.065
Unknown	42(0.59)	69(0.97)	58(0.82)	51(0.72)	
Primary school and below	4796(67.35)	5136(72.14)	5188(72.91)	5093(71.55)	
Junior/senior high school	2215(31.11)	1848(25.96)	1769(24.86)	1890(26.55)	
College and above	68(0.95)	66(0.93)	101(1.42)	84(1.18)	
Drinking (%)	1677(23.55)	1542(21.66)	1676(23.55)	1485(20.86)	**0.027**
Smoking (%)	1641(23.04)	1402(19.69)	1432(20.12)	1457(20.47)	**0.001**
BMI (kg·m^−2^)	24.8(22.8, 27.0)	24.9(22.8, 27.1)	24.9(22.8, 27.0)	25.0(22.9, 27.3)	0.205
SBP (mmHg)	163(149, 172)	162(142, 171)	162(142, 170)	161(141, 169)	**0.001**
DBP (mmHg)	89(81, 98)	88(80, 97)	89(80, 98)	88(79, 96)	0.133
Heart rate (beats/min)	76(70, 84)	76(70, 84)	76(70, 84)	76(69, 83)	**0.002**
TC (mmol/L)	5.03(4.28, 6.03)	5.09(4.29, 6.19)	5.01(4.22, 6.11)	5.22(4.36, 6.46)	0.630
LDL (mmol/L)	2.72(2.03, 3.33)	2.87(2.13, 3.60)	2.85(2.13, 3.57)	2.94(2.25, 4.11)	0.831
HDL (mmol/L)	1.47(1.15, 1.84)	1.42(1.11, 1.77)	1.39(1.09, 1.73)	1.41(1.09, 1.75)	0.525
TG (mmol/L)	1.76(1.18, 2.68)	1.69(1.13, 2.61)	1.68(1.12, 2.52)	1.70(1.15, 2.57)	0.459

Abrreviations: CVD, cardiovascular disease; BMI, body mass index; SBP, systolic blood pressure; DBP, diastolic blood pressure; TC, total cholesterol; LDL, high-density lipoprotein; HDL, high-density lipoprotein; TG, triglycerides.

Q_1_:lowest adherence; Q_2_:second adherence; Q_3_:third adherence; Q_4_:highest adherence.

### 3.4 Relationship between dietary pattern and CVD

Table 7 presents the results of the Cox regression analysis of the relationship between different dietary patterns and CVD risk. Analysis revealed significant correlations between adherence to the "Bean, egg, milk and pickle" dietary pattern and CVD risk. This association remained significant after adjusting for confounders. After full adjustment (Model 3), the risk of CVD in the highest quartile of adherence increased by 29% compared to the lowest quartile (HR = 1.29; 95% CI:1.09, 1.54; p = 0.001). Additionally, the "Seafood and animal meat" dietary patterns was associated with an increased risk of CVD (HR = 1.22; 95% CI:1.04, 1.44; p = 0.028).However, after full adjustment for confounders, this risk slightly decreased (HR = 1.16; 95% CI:0.99, 1.37; p = 0.116), while the association became non-significant. In the highest quartile of the initial model (model 1), adherence to the "Wheat and whole grains" dietary pattern was associated with a 21% reduction in CVD risk (HR = 0.79; 95%CI: 0.29, 2.14; p = 0.595), but this result was not statistically significant. Following complete adjustment for confounders, the risk increased(HR = 1.07; 95% CI:0.19, 1.26; p = 0.560), with no significant correlation observed. Lastly, there was no significant association between adherence to the "Rice and vegetable" dietary pattern and CVD risk (HR = 1.06; 95% CI:0.90, 1.24; p = 0.644).

**Table 7:** Cox regression analysis of the relationship between different dietary patterns and CVD risk.

Characteristic		Cases (n)	Incidence density(1/1000PYs)	HR(95%CI)		
				Model 1	Model 2	Model 3
Bean, egg, milk and pickle	Q_1_	259	6.78	1.00	1.00	1.00
	Q_2_	292	7.22	1.16(0.98, 1.38)	1.16(0.98, 1.38)	1.15(0.98, 1.37)
	Q_3_	305	7.80	**1.31(1.11, 1.56)∗**	**1.31(1.10, 1.55)∗**	**1.30(1.09, 1.53)∗**
	Q_4_	318	7.75	**1.30(1.10, 1.54)∗**	**1.28(1.08, 1.53)∗**	**1.29(1.09, 1.54)∗**
	p _trend_			**0.001**	**0.001**	**0.001**
Seafood and animal meat	Q_1_	252	6.16	1.00	1.00	1.00
	Q_2_	298	7.35	1.09(0.92, 1.29)	1.08(0.91, 1.28)	1.06(0.90, 1.26)
	Q_3_	277	7.35	1.05(0.88, 1.24)	1.03(0.87, 1.23)	1.01(0.85, 1.20)
	Q_4_	347	8.77	**1.22(1.04, 1.44)∗**	**1.26(1.02, 1.42)∗**	1.16(0.99, 1.37)
	p _trend_			**0.028**	**0.043**	0.116
Wheat and coarse cereals	Q_1_	301	7.47	1.00	1.00	1.00
	Q_2_	309	7.95	1.02(0.53, 1.98)	1.06(0.90, 1.24)	1.05(0.89, 1.23)
	Q_3_	276	6.94	0.86(0.44, 1.69)	1.00(0.85, 1.18)	1.00(0.85, 1.18)
	Q_4_	288	7.23	0.79(0.29, 2.14)	1.07(0.90, 1.26)	1.07(0.91, 1.26)
	p _trend_			0.595	0.574	0.560
Rice and vegetable	Q_1_	303	7.49	1.00	1.00	1.00
	Q_2_	290	7.27	0.99(0.84, 1.16)	1.00(0.85, 1.17)	1.01(0.86, 1.19)
	Q_3_	271	6.91	0.94(0.80, 1.11)	0.95(0.81, 1.12)	0.97(0.82, 1.14)
	Q_4_	310	7.91	1.02(0.87, 1.20)	1.03(0.87, 1.20)	1.06(0.90, 1.24)
	p _trend_			0.962	0.909	0.644

∗: p < 0·05. Model 1: adjusted age, gender, urban-rural division and education level; Model 2: adjusted model 1+drinking and smoking; Model 3: adjusted model 2+SBP and heart rate.

In summary, adherence to the "Bean, egg, milk and pickle" dietary patterns was positively associated with the incidence of CVD and this association persisted after full adjustment for confounding factors. Conversely, adherence to the "Seafood and animal" dietary pattern, "Wheat and whole grains" dietary pattern, and "Rice and vegetable" dietary patterns was not significantly associated with CVD risk.

### 3.5 Sensitivity analysis

**Fig. 1:**
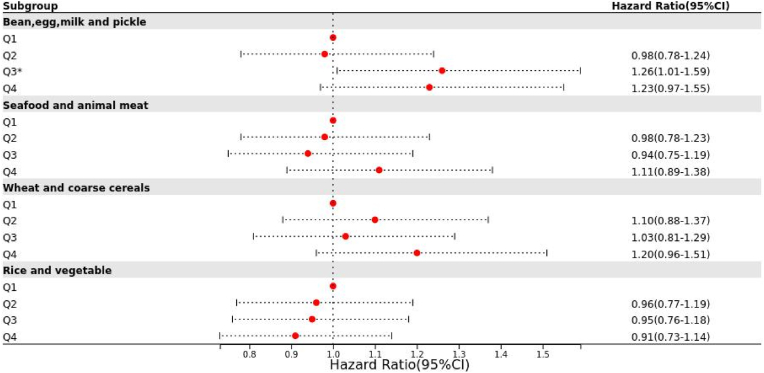
Sensitivity analysis of the relationship between dietary patterns and CVD incidence.

A sensitivity analysis was conducted by excluding people whose follow-up period was less than two years, using Model 3 for verification. The results, illustrated in Fig. 1, revealed that adherence to the "Bean, egg, milk and pickle" dietary pattern in the third quartile remained significantly associated with an increased risk of CVD (HR = 1.26; 95% CI:1.01, 1.59; p = 0.045). No other dietary patterns demonstrated significant associations with CVD risk. These findings indicate that the results are robust and reliable.Furthermore, the risk of cardiovascular disease among high-risk indiviuals does not appear to be biased towards a specific dietary pattern but may be influenced by other confounding factors.

## 4 Discussion

Improving lifestyle, particularly dietary habits, is an economical and highly acceptable way to prevent and control CVD, with dietary structure being a critical aspect (Smith et al., 2013). This study aimed to elucidate the relationship between dietary structure and the incidence of CVD in high-risk groups. According to the results of factor analysis, four main dietary patterns were identified: "Bean, egg, milk and pickle", "Seafood and animal meat", "Wheat and coarse cereals", and "Rice and vegetable", explaining 56.87% of the total variance in dietary data. In a fully adjusted model, adherence to the "Bean, egg, milk and pickle" dietary pattern was associated with a increase risk of CVD. Compared with the lowest quartile, the risk in the highest quartile population increased by 29%(p = 0.001). Adherence to the "Seafood and animal meat" dietary pattern, "Wheat and coarse cereals" dietary pattern, and the "Rice and vegetable" dietary pattern showed no significant impact on the outcome of CVD. The sensitivity analysis revealed an association with increased CVD risk only in the third quartile of the of adherence to the "Bean, egg, milk and pickle" dietary pattern (HR = 1.26; 95% CI: 1.01, 1.59; p = 0.045), while no other dietary patterns showed significant associations with cardiovascular disease. This study comtributes to the limited literature by employing factor analysis to assess the association between dietary structures and CVD risk, indicating that specific dietary patterns may not represent a potential risk for disease among high-risk populations. These findings have important implications for future research. In the future, employing diverse dietary pattern evaluation methods will allow for comparisons of dietary patterns established by different approaches within the same population. This comprehensive analysis could enhance dietary guidance and provide a stronger foundation for the prevention and management of CVD in high-risk groups.

A previous 10-year cohort study consisted of approximately 500,000 Chinese residents aged 30–79 years who had no history of cardiovascular disease and identified dietary patterns (the modern dietary pattern) similar to those in this study (Qin et al., 2021). In that cohort, the "modern" dietary pattern, characterized by high consumption of fresh fruits, meat, poultry, fish, dairy products and soybeans, is comparable to the "Bean, egg, milk and pickle" dietary pattern observed in this study. The "modern" dietary pattern is associated with a reduced risk of cardiovascular disease rather than other risk factors. The "modern" dietary pattern includes high-loading components such as fruit, beans, poultry and milk, which are also key contributors to the Mediterranean and DASH diet. However, the "Bean, egg, milk and pickle" dietary pattern identified in this study was positively correlated with CVD. Notably, the "Bean, egg, milk and pickle" dietary pattern had a high loading of pickles, which have been positively associated with cardiometabolic diseases in several studies (Mozaffarian, 2016). It is plausible that the potentially harmful effects of pickles, particularly their high salt content, may outweigh the beneficial contributions of beans, eggs, and milk within this dietary pattern. This finding suggests that certain component, such as the elevated sodium levels in pickles, could mitigate the overall protective effects typically associated with healthier dietary patterns. In a US population-based study involving 29,682 participants, Zhong et al. found that a higher intake of processed meat or poultry was significantly associated with a slightly increased risk of CVD events (Zhong et al., 2020). Similarly, a large multinational prospective study involving 134,297 participants by Iqbal et al. found that intake of processed meat was positively correlated with an increased risk of major CVD (Iqbal et al., 2021). In contrast, a meta-analysis of 6 observational studies involving 1,330,352 individuals and137,376 deaths indicated that unprocessed red meat was not associated with an increased risk of mortality (Larsson and Orsini, 2014). Most studies that have reported adverse associations were from the Western countries, whereas no significant association was observed in studies conducted in Asia (Lee et al., 2013)^.^ Similarly, in our study, although the "Seafood and animal meat" dietary pattern was associated with an increased risk of disease, no significant correlation was found.

In a large prospective cohort study involving 13,055 adults followed for a median of 9 years, 502 participants were found to develop CVD. This study on the Chinese population revealed that a traditional dietary pattern characterized by a high intake of rice, pork, fish, poultry, and fresh vegetables, but a low intake of wheat was associated with a low risk of CVD. Interestingly, rice intake was inversely associated, while wheat intake was directly associated with CVD risk (Shi et al., 2020). These findings align with previous cross-sectional findings in the China Health and Nutrition Survey (CHNS) and other studies in China (Ye et al., 2018; Sun et al., 2013). These findings were also supported by the current knowledge on the relationship between dietary patterns and CVD risk (Shi et al., 2012, 2017). The traditional dietary pattern, characterized by a high intake of rice, pork, and fresh vegetables, resembles the dietary pattern observed in this study for rice and vegetables. However, in our study, this dietary pattern was not associated with CVD, which may be attributed to differences in the high-risk population under investigation.

The innovation of this study lies in its comprehensive analysis of dietary patterns among a high-risk population for CVD aged 35 and older. This is the first study to focus on this demographic, utilizing a large sample size and encompassing a wide age range, while thoroughly adjusting for both established and potential risk factors. These strengths provide a scientific basis for guiding dietary patterns among high-risk groups and for the prevention and control of CVD. However, the study has several limitations. First, since the participants primarily reside in the Zhejiang region, the findings may not be generalizable to other regions of China or to other provincial populations, necessitating further research. Second, the FFQ used was self-reported, which could lead to recall bias and potential misclassification affecting the factor load in the dietary patterns. Additionally, the FFQ only measured dietary intake at baseline, leaving uncertainty about whether participants’ dietary patterns changed during the follow-up period. The study used only 12 major food groups to construct the dietary patterns, potentially omitting other important dietary habits, such as the consumption of fried or sweet foods (Sisa et al., 2021). Finally, residual confounding may still have biased the observed correlations despite adjustments for multiple covariates.

## 5 Conclusions

This study indicates that a higher intakes of beans, eggs, milk, and pickles is significantly associated with a modest increase in CVD risk among individuals over the age of 35 who are at high risk of CVD in China. While the findings indicate that only the "Bean, Egg, Milk, and Pickle" dietary pattern is significantly linked to CVD risk, the study contributes valuable insights into the relationship between dietary patterns and disease risk in high-risk populations. Future studies should investigate the relationship between changes in dietary patterns and CVD risk over time to provide a robust scientific basis for dietary guidance tailored to high-risk groups.

## CRediT authorship contribution statement

**Shanshan Chen:** conceived the idea, analysed data, interpreted results, and, drafted the orginal manuscript. **Shiyun Hu:** conceived the idea, managed data curation. **Sijie Shen:** revised the manuscript. **Jialin Zhang:** re-analysed the data and validated results. **Xiaohui Xu:** re-analysed the data and validated results. **Ming Yu:** supervised the study. **Yu Xia:** supervised the study. **Qiang Cai:** were responsible for, Funding acquisition, and, Supervision, All authors critically reviewed and approved the final version of the manuscript. **Wei Yu:** conceived the idea, were responsible for, Funding acquisition, and, Supervision. **Anni Lu:** revised the manuscript. **Ziqi Mia Li:** revised the manuscript. **Rasika Gunarathne:** revised the manuscript. **Jun Lu:** conceived the idea, were responsible for, Funding acquisition, and, Supervision, provided professional advice on the cohort study design and reviewed and edited the final draft.

## Ethics statement

Ethical approval and Consent to Participate: This study was approved by the Zhejiang Hospital Medical Ethics Committee (No 27 K, 2019). All participants provided their informed consent in writing, either by thumbprint or signature.

## Funding

This work was funded by the Zhejiang Provincial Medical and 10.13039/100018696Health Major Science and Technology Program [2018277576] and 10.13039/501100017599Major Science and Technology Projects of Zhejiang Province [2014C03045-1].

## Declaration of competing interest

The authors declare that they have no known competing financial interests or personal relationships that could have appeared to influence the work reported in this paper.

## Data Availability

Data will be made available on request.
